# In Silico Functional and Structural Analysis of *STAT4* Variants of Uncertain Significance

**DOI:** 10.3390/genes17010072

**Published:** 2026-01-07

**Authors:** Karla Mayela Bravo-Villagra, Eric Jonathan Maciel-Cruz, Rosa Michel Martínez-Contreras, Itzae Adonai Gutiérrez-Hurtado, Alexis Missael Vizcaíno-Quirarte, José Francisco Muñoz-Valle, Andres López-Quintero

**Affiliations:** 1 Centro Universitario de Ciencias de la Salud, Instituto de Nutrigenética y Nutrigenómica Traslacional, Universidad de Guadalajara, Guadalajara 44340, Jalisco, Mexico; karla.bravo2318@alumnos.udg.mx; 2 Programa de Doctorado en Genética Humana, Centro Universitario de Ciencias de la Salud, Universidad de Guadalajara, Guadalajara 44340, Jalisco, Mexico; 3 Centro de Investigación Biomédica de Occidente, Instituto Mexicano del Seguro Social, Guadalajara 44340, Jalisco, Mexico; 4 Departamento de Biología Molecular y Genómica, Centro Universitario de Ciencias de la Salud, Universidad de Guadalajara, Guadalajara 44340, Jalisco, Mexico; 5 Centro Universitario de Ciencias de la Salud, Instituto de Investigación en Ciencias Biomédicas, Universidad de Guadalajara, Guadalajara 44340, Jalisco, Mexico; 6 Programa de Doctorado en Psicología de la Salud, Centro Universitario de Ciencias de la Salud, Universidad de Guadalajara, Guadalajara 44340, Jalisco, Mexico

**Keywords:** *STAT4* gene, variants of uncertain significance (VUS), in silico analysis, bioinformatics tools, pathogenicity prediction

## Abstract

Background: The *STAT4* gene plays a key role in immune regulation and is associated with susceptibility to autoimmune diseases such as rheumatoid arthritis (RA) and systemic lupus erythematosus (SLE). Objectives: The objective of this study is to analyze variants of uncertain significance (VUSs) in *STAT4* using bioinformatics tools to predict their functional and structural impact. Methods: A total of 48,295 variants of the *STAT4* gene (ENSG00000138378) were retrieved from the Ensembl database. A tiered filtering approach was used to assess VUS pathogenicity, integrating in silico prediction tools such as SIFT, PolyPhen, MutPred2, and Align-GVGD, as well as structural modeling platforms including Chimera, ModRefiner, Missense3D, HOPE, and DynaMut2. Results: Eighty missense VUSs were identified; of these, 13 were prioritized based on concordant signals across multiple computational predictors. These variants showed significant alterations in the physicochemical properties of the protein, including changes in hydrophobicity and disruption of hydrogen bonding. Notably, the rs140675301 (Glu128Val) variant lies within a conserved loop, and in silico analyses suggest that this mutation may alter kinase specificity regarding the phosphorylation of serine 130. Conclusions: The integrative use of the bioinformatic tools employed represents a valuable preliminary step prior to undertaking more complex and resource-intensive functional studies. This complementary strategy strengthens the interpretative framework for VUS, guiding subsequent experimental validation and supporting a structured assessment of variant relevance, particularly in the context of immune-related genes such as *STAT4*.

## 1. Introduction

Autoimmune diseases are complex and multifactorial disorders characterized by an inappropriate immune response against the body’s own components. Genomic studies have identified multiple genetic susceptibility loci, among which genes from the major histocompatibility complex (*HLA*) stand out due to their central role in antigen presentation [1,2]. However, several non-*HLA* genes involved in immune regulation have also been identified. Among these, *STAT4* has been widely associated with increased susceptibility to various autoimmune diseases, highlighting its relevance as a genetic factor of interest in the study of these conditions [3,4,5,6,7].

The *STAT4* gene, located on chromosome 2 at cytogenetic band *2q32.2*, encodes the signal transducer and activator of transcription 4 (*STAT4*) protein [8]. This gene comprises 24 exons that encode various functional domains of the protein, including the DNA-binding and transactivation domains [9,10].

As a member of the STAT family, STAT4 plays a pivotal role in immune system regulation and has been implicated in a range of autoimmune and inflammatory diseases [11,12]. STAT4 activation occurs via the JAK-STAT signaling pathway; once phosphorylated, the protein translocates to the nucleus, where it regulates the expression of genes involved in immune responses [8,11,12].

Several single-nucleotide variants (SNVs) in *STAT4* have been associated with increased susceptibility to diseases such as RAand SLE. These variants have also been linked to higher disease activity in RA and elevated levels of anti-cyclic citrullinated peptide (anti-CCP) antibodies [5,13]. These findings underscore the importance of *STAT4* as a shared genetic risk factor across multiple immune-mediated diseases [3]. However, the interpretation of VUS in the *STAT4* gene remains a significant challenge in medical genetics, as such variants may influence disease risk and therapeutic responses [14]. Therefore, this study aimed to apply bioinformatics tools to predict the pathogenic potential of these variants using a systematic filtering and analysis approach.

## 2. Materials and Methods

### 2.1. In Silico Prediction of STAT4 Variants

The in silico prioritization of *STAT4* VUS was conducted using a tiered filtering approach, integrating several in silico platforms categorized by similar predictive properties. A variant was retained for further analysis only if it was flagged as potentially impactful by more than one tool.

### 2.2. Data Collection and Variant Filtering

Information on the *STAT4* gene was obtained from Ensembl (ENSG00000138378) (https://www.ensembl.org/info/index.html) accessed on 26 September 2025 [15,16]. The data were processed in Microsoft Excel, and variants were selected based on the criteria: “SNP,” “missense,” “uncertain significance,” and “dbSNP source.” A total of 80 VUSs were identified for computational analysis. The STAT4 canonical protein sequence (Q14765) was retrieved in FASTA format from UniProt (https://www.uniprot.org) accessed on 26 September 2025 [17].

### 2.3. Functional and Structural Predictions of Variants

In the initial screening phase, all 80 variants were analyzed to assess their potential functional impact, particularly with respect to structural and regulatory relevance. Predictive tools such as SIFT (https://sift.bii.a-star.edu.sg/www/SIFT_dbSNP.html) accessed on 13 October 2025 were employed to evaluate whether amino acid substitutions might impair protein function. SIFT assigns scores between 0 and 1; values below 0.05 indicate a likely deleterious effect [18]. PolyPhen (http://genetics.bwh.harvard.edu/pph2/) accessed on 13 October 2025 [19] was used to assess physicochemical differences between wild-type and substituted residues, with scores above 0.5 suggesting probable functional disruption.

### 2.4. Evaluation of Protein Stability

Fifty-four variants underwent further evaluation using Align GVGD and MutPred2. Align GVGD (http://agvgd.hci.utah.edu/index.php) accessed on 14 October 2025, integrates evolutionary conservation and biochemical properties to classify variants on a scale from C0 to C65, where C15–C65 are suggestive of potential functional impact [20,21]. MutPred2 (http://mutpred2.mutdb.org/index.html) accessed on 15 October 2025 evaluates structural and functional impacts, providing scores from 0 to 1. Scores above 0.51 are indicative of potential structural or functional relevance [22].

### 2.5. Structural Protein Modeling

Structural models of the 54 filtered variants were generated using Chimera, ModRefiner, ERRAT, and PROCHECK. Chimera (https://www.cgl.ucsf.edu/chimera/) accessed on 16 October 2025, was used to visualize three-dimensional molecular changes [23]. ModRefiner (https://zhanggroup.org/ModRefiner/) accessed on 16 October 2025 [24] refined structural geometry. The quality of the models was further assessed by ERRAT and PROCHECK (https://saves.mbi.ucla.edu/) accessed on 16 October 2025, which evaluate stereochemical accuracy based on empirical data [25].

### 2.6. Structural Impact of Variants

Missense3D (http://missense3d.bc.ic.ac.uk/missense3d/) accessed on 17 October 2025 was used to predict the 3D structural consequences of missense variants [26]. In parallel, HOPE (https://www3.cmbi.umcn.nl/hope/) accessed on 18 October 2025 provided detailed information about physicochemical changes (e.g., size, charge, polarity) and their potential effects on protein structure and function [27]. A total of 54 variants were assessed with these tools.

### 2.7. Molecular Dynamics and Structural Stability

Based on predictions from HOPE and Missense3D, 13 variants were selected for molecular dynamics analysis using DynaMut (https://biosig.lab.uq.edu.au/dynamut/) accessed on 19 October 2025 [28]. Structural stability was evaluated using the ΔΔG scale: values < 0 indicate destabilization, values > 0 indicate stabilization, and values = 0 suggest no significant effect.

### 2.8. Statistical Analysis

Statistical analyses were performed using R and RStudio (version 4.4.1). The Shapiro–Wilk test was applied to assess the normality of the ΔΔG data. Data dispersion was visualized using boxplots, and correlations among variables were depicted using heatmaps. A *p*-value of <0.05 was considered statistically significant.

### 2.9. Workflow Diagram

**Figure 1:**
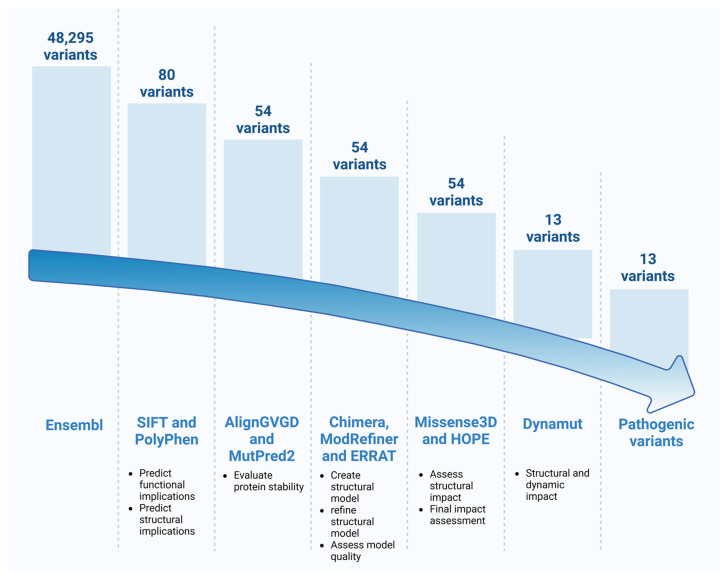
Diagram of Bioinformatics Tool Workflow for Variant Prediction. Eighty missense variants of uncertain significance were identified from the Ensembl database. Predictive tools included SIFT, PolyPhen2, MutPred2, and Align GVGD. Structural and molecular analyses were performed using Chimera, ModRefiner, Missense3D, HOPE and DynaMut2. Image created with BioRender.

A comprehensive workflow summarizing the bioinformatics tools and sequential steps used in the variant prediction pipeline is presented in Figure 1, detailing the platforms applied at each stage to assess the potential structural and functional impact of the selected *STAT4* variants.

## 3. Results

A total of 48,295 variants of the *STAT4* gene (ENSG00000138378) were retrieved from the Ensembl database. From these, 80 missense VUSs were selected for downstream analysis based on the criteria described. These variants were processed using a structured bioinformatics workflow, applying multiple in silico prediction tools grouped according to their methodological scope.

### 3.1. Functional and Structural Predictions of Variants

Of the 80 variants analyzed, SIFT predicted 34 variants (42.5%) as tolerated and 46 variants (57.5%) as deleterious, while PolyPhen2 predicted 42 variants (52.5%) as benign and 38 (47.5%) as possibly damaging. Concordance between both platforms identified 26 variants with consistent benign predictions, whereas 34 variants showed concordant damaging predictions. Notably, 20 variants exhibited discordant classifications, highlighting variability among functional predictors and supporting their use for variant prioritization rather than definitive inference.

### 3.2. Evaluation of Protein Stability

Following the initial screening, 54 variants were subjected to further analysis using Align GVGD and MutPred2. Align GVGD categorized 3 variants (5.55%) as benign and 51 (94.44%) showed non-benign grades, indicating potential functional relevance. MutPred2 predicted 23 variants (42.59%) as low-risk and 32 variants (59.26%) as high-risk based on its scoring framework. Integration of predictions across platforms revealed that 2 variants were flagged by a single tool, 14 variants by two tools, 12 variants by three tools, and 26 variants were consistently flagged across all four platforms, supporting their prioritization for downstream structural analysis.

### 3.3. Evaluation of the Structural Impact of Variants

HOPE and Missense3D were applied to the complete set of 54 missense variants to evaluate their potential structural impact on the STAT4 protein. Based on a harmonized interpretation of the structural outputs, 41 variants (75.9%) showed no consistent evidence of structural damage, whereas 13 variants (24.1%) were prioritized as candidates due to the presence of physicochemical changes and/or structural signals reported by at least one of the predictors used.

**Figure 2:**
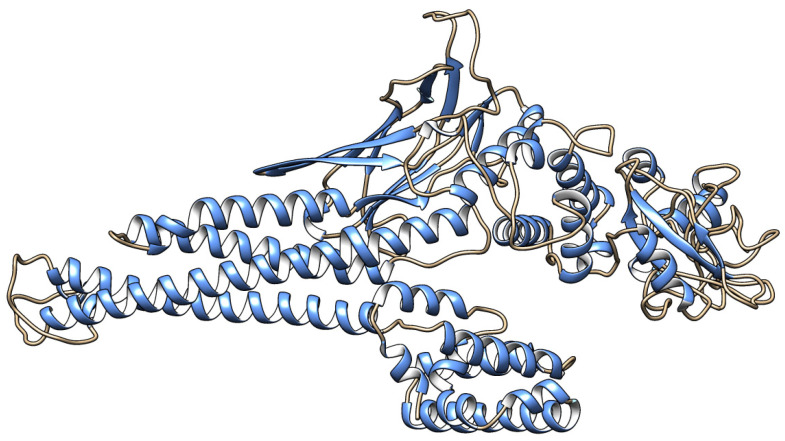
Three-dimensional representation of the STAT4 protein structure. The molecular structure of the STAT4 protein was modeled and visualized using Chimera. Structural alterations resulting from selected missense variants were assessed in relation to their spatial localization and potential impact on conserved domains.

Complementary analysis using HOPE provided detailed descriptions of the physicochemical changes associated with each variant, while Missense3D and DynaMut2 contributed structural damage indicators and protein stability estimates, respectively. Importantly, these results are intended for variant prioritization and in silico hypothesis generation, and not for inferring biological pathogenicity. A summary of these findings is presented in Table 1 and visualized in Figure 2.

### 3.4. Molecular Dynamics and Structural Stability Analysis

To further explore the potential structural consequences of amino acid substitutions, a molecular dynamics-based approach was applied using DynaMut2. Stability predictions were derived from changes in Gibbs free energy (ΔΔG), providing estimates of the relative impact of each substitution on protein stability.

**Figure 3:**
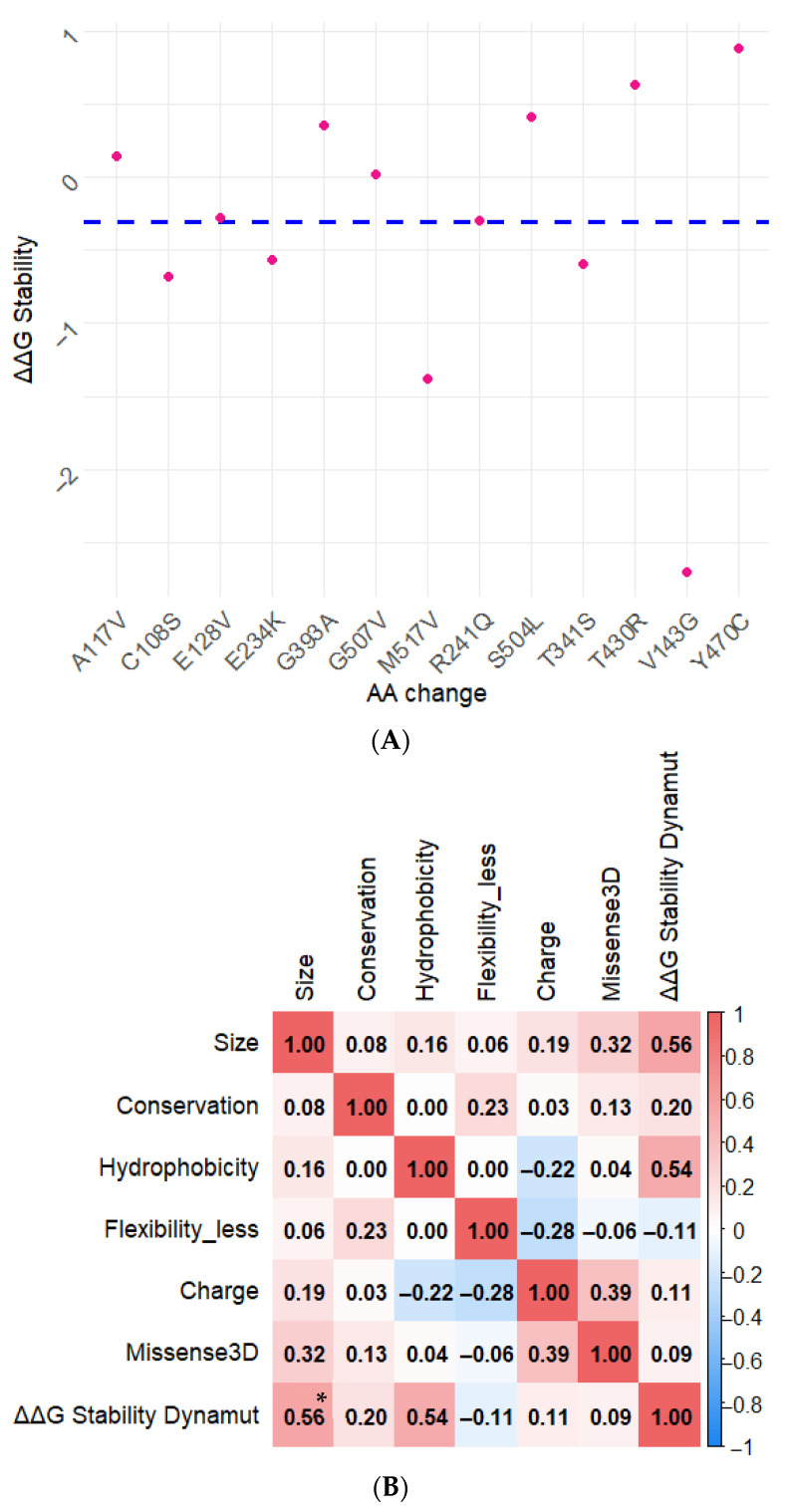
Structural Impact and Correlation Analysis of STAT4 Missense Variants. (**A**) Scatter plot showing the predicted impact of amino acid substitutions on protein stability (ΔΔG values) as determined by DynaMut2. Each point represents a missense variant, with the dashed line indicating the mean ΔΔG value, serving as a reference for assessing relative destabilization effects. (**B**). Heatmap illustrating the correlation matrix between seven structural and physicochemical variables. Numerical values within each cell represent Spearman’s correlation coefficients. Color intensity denotes the strength and direction of the correlation, with red indicating positive and blue negative associations. * A statistically significant positive correlation was observed between the amino acid size change and ΔΔG stability (ρ = 0.56, *p* < 0.05).

Thirteen variants prioritized through the integrated in silico workflow were analyzed (Table 1). Most variants showed negative ΔΔG values, suggesting a tendency toward reduced structural stability (Figure 3A). Additionally, correlation analysis incorporating structural and physicochemical variables derived from HOPE and Missense3D revealed a statistically significant association between changes in amino acid size and ΔΔG values (Figure 3B), indicating that charge alterations may influence predicted stability effects.

### 3.5. Validation of In Silico Predictive Tools

To strengthen the methodological framework of this study, a comparative analysis was performed using seven additional variants of the *STAT4* gene previously reported and classified in clinical databases as benign (B), likely benign (PB), possibly pathogenic (PP), or pathogenic (P). This validation allowed us to assess the concordance between the in silico predictions obtained through SIFT, PolyPhen-2, Align-GVGD, MutPred2, Missense3D, HOPE, and DynaMut, and the clinical classifications based on ACMG criteria (see Supplementary Materials Tables S1 and S2).

Among the seven variants analyzed, only rs35279173 maintained full concordance with its benign classification across all predictors. In the remaining six variants, discrepancies were observed between computational predictions and clinical classifications, highlighting the limitations of these tools in reproducing the true functional impact. For instance, rs2470644108 and rs2470644386, which are clinically classified as pathogenic, were estimated by DynaMut and Missense3D as stabilizing variants, whereas HOPE identified significant alterations in residue charge and in the surrounding domain environment. Similarly, variants classified as PB and PP (rs61756200, rs3024839, and rs2470644258) displayed negative ΔΔG values, suggesting a possible destabilizing effect on protein structure and showing relevant physicochemical changes in conserved regions.

## 4. Discussion

VUS represents a major challenge in genetic counseling and clinical decision-making due to the difficulty in interpreting their functional impact. In this context, in silico analysis of VUSs that lead to amino acid substitutions can offer valuable insights into potential structural and functional consequences, supporting efforts toward variant prioritization and hypothesis generation, rather than definitive reclassification [29].

Several studies have underscored the limitations in VUS interpretation. While in vitro experiments provide highly accurate functional characterization, they are resource-intensive and capable of evaluating only a limited number of variants [30,31]. In silico methodologies have therefore emerged as a complementary approach, offering predictive assessments often based on evolutionary conservation and biophysical modeling [29,30,32]. Recent advances in artificial intelligence have further accelerated variant interpretation, with structure-based models showing promising performance [31,33]. However, these predictors should be used with caution. Several studies have documented frequent discrepancies between the results obtained through computational predictions and the data obtained through in vitro validations, highlighting the importance of integrating multiple lines of evidence [31].

Tools such as SIFT and PolyPhen-2 employ distinct algorithms: SIFT uses nonsynonymous single-nucleotide variants to calculate the probability of each substitution (SIFT score) [34], while PolyPhen-2 relies on eight sequence-based and three structural features as inputs for a Bayesian classifier. These methodological differences may limit their clinical applicability in classifying VUS as pathogenic [18,35]. On the other hand, Align-GVGD uses logistic regression divided into four categories and is based exclusively on physicochemical changes resulting from amino acid substitutions relative to observed variability, providing a certain robustness to the evidence it generates [36]. MutPred2 is a machine learning model trained on unlabeled data that estimates probabilities, allowing the modeling of the impact of variants on protein structure and function while assigning potential molecular effects. It has proven useful for predicting de novo mutations that are more frequently pathogenic in cases than in controls [22].

Similarly, the HOPE, Missense3D, and DynaMut tools are valuable for predicting the structural and functional impact of variants. HOPE (web-based server, accessed on 18 October 2025) employs BLAST-formatted sequences, followed by automated homology modeling, and finally analyzes the characteristics of wild-type and mutant amino acids through web services [27]. Missense3D focuses on identifying three-dimensional structural changes in the protein, performing predictions through its web server [26], whereas DynaMut2, through a programming-based framework with defined input and output variables, estimates stability changes using molecular dynamics simulations to model the atomic behavior of the protein [28]. Given the variability of their underlying mechanisms—ranging from evolutionary conservation and physicochemical properties to structural modeling and dynamic simulation—each tool contributes a unique layer of evidence. Therefore, relying on a single predictive model is insufficient for robust variant classification. Instead, integrating multiple computational tools allows for a more comprehensive assessment of possible functional consequences.

In this study, 80 missense VUSs in the *STAT4* gene were identified and systematically evaluated. Thirteen of these (16.25%) were prioritized based on concordant signals across multiple predictors [37], reflecting physicochemical changes such as alterations in amino acid size, hydrophobicity, or charge, and their localization within or near conserved regions. Accumulating evidence suggests that surface hydrophobicity and residue charge play important roles in protein folding and structural integrity [38]. Disruptions in these properties may influence hydrogen bonding networks, local conformational stability, and protein dynamics [39]. Nevertheless, these observations should be interpreted as structural hypotheses rather than direct evidence of functional impairment.

Despite these in silico predictions, the ACMG classification framework [40] categorized all 13 variants as either benign or of uncertain significance. This divergence may be attributed to the lack of supporting functional or association data, often due to the extremely low allele frequencies of these variants (global minor allele frequency < 0.01), which limits the statistical power of existing datasets to confirm pathogenicity. Population frequency data were considered at a global level, and no population-stratified analyses were performed; therefore, population-specific inferences were beyond the scope of this study.

**Figure 4:**
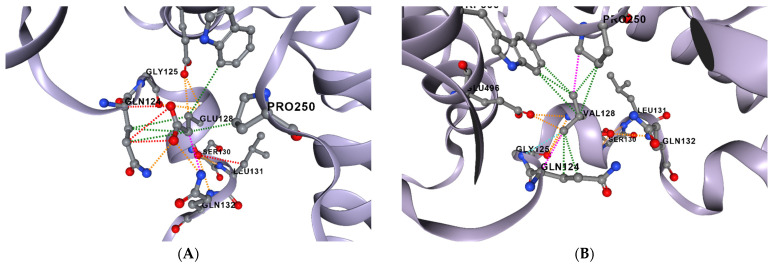
Structural impact of the Glu128Val mutation in the STAT4 protein. (**A**) Wild-type structure highlighting the glutamic acid (Glu) at position 128. (**B**) Mutant structure displaying the valine (Val) substitution at the same position. The mutation results in changes in charge, size, and hydrophobicity of the residue, potentially affecting local structural integrity and phosphorylation dynamics. Protein modeling and visualization were performed using DynaMut2.

Among the 13 analyzed variants, rs140675301 (Glu128Val) illustrates how structural modeling can inform hypothesis generation. This substitution involves the replacement of a negatively charged glutamic acid with a hydrophobic valine, altering the charge, hydrophobicity, and size of the residue. Structural modeling revealed that the variant is located within a surface-exposed, conserved loop, in proximity to a putative phosphorylation site at Ser130. In silico predictions suggest that this substitution may influence kinase recognition or local phosphorylation dynamics, highlighting a potential regulatory mechanism that warrants further functional investigation, rather than confirming a pathogenic role [41,42]. These predicted features are illustrated in Figure 4.

Analysis of molecular dynamics outputs from DynaMut2, integrated with structural descriptors derived from HOPE and Missense3D, further emphasized the contribution of amino acid size and hydrophobicity changes to predicted stability effects [43]. However, these predictions remain probabilistic and context-dependent, underscoring the limitations of computational tools in capturing complex three-dimensional and interaction-specific effects.

To strengthen the methodology, we validated seven missense variants classified as B, LB, LP, and P. Only the variant rs35279173 maintained full concordance with its benign classification across all predictors. The impact of amino acid substitutions on protein function is multifactorial and depends on the specific location of the residue within the protein structure, its involvement in molecular interactions, and its role in regulatory mechanisms [41]. Modifications at critical positions may result in either loss or gain of function and could interfere with interactions involving other molecules, ultimately influencing regulation and stability of the protein [8,14]. These findings emphasize that structural and functional predictors do not always capture three dimensional or interaction effects, which may result in falsely neutral predictions. Integrating multiple computational tools and balancing evolutionary and structural information is recommended for more accurate variant classification.

A major limitation of this study is the absence of experimental validation or association analyses to directly assess biological impact. While computational approaches offer valuable insights, their findings must be validated through in vitro or in vivo experiments to confirm their biological significance. Nevertheless, the present work offers a reproducible methodological framework for variant prioritization, identifying candidates for downstream functional studies and contributing to a more structured interpretation of VUS in *STAT4*.

## 5. Conclusions

In this study, an integrative in silico variant prioritization framework was applied to missense variants of the *STAT4* gene, allowing the identification of thirteen variants prioritized based on predicted structural and physicochemical features. The potential impact of these variants is influenced by their location within specific protein domains and by the nature of the amino acid substitutions involved. Variants such as p.E128V, located near a known phosphorylation site, illustrate how structural modeling can generate testable hypotheses regarding possible effects on protein regulation. However, these observations should not be interpreted as evidence of biological pathogenicity. Overall, this work highlights the value of combining multiple computational tools to support the interpretation and prioritization of VUS. While functional validation remains essential, the proposed framework provides a reproducible methodological approach that can be extended to other genes and datasets in future studies. In this context, the proposed workflow may support genetic counseling and translational research by providing a structured approach to prioritize VUS for targeted functional testing and follow-up, without replacing clinical or experimental evaluation.

## Figures and Tables

**Table 1:** Structural Prediction Analysis of 13 Missense Variants in *STAT4* using HOPE, Missense3D, and DynaMut2.

						HOPE	Missense 3D	Dynamut2
Variant	Allele Frequencies	Amino Acid Change	ACMG	VarSome	Score	Main Prediction	Main Prediction	Predicted Stability Change (ΔΔG)
	(gnomAD)				MetaRNN			
rs755317297	T: 1.000	M517V	VUS	VUS	0.843	Smaller, mutant residue located near a highly conserved region. The mutant residue does not prefer α-helices as secondary structure	No structural damage detected	−1.379
	A: 6.841 ×10^−7^			B				
	C: 2.736 × 10^−6^							
rs1192576162	C: 0.999992036	G507V	VUS	P	0.77	Bigger, more hydrophobic, residue is located near a conserved region	Glycine in Bend Buried Glycine Replaced	0.021
	A: 7.96400 × 10^−6^			VUS				
	
rs1235014939	G: 0.999992036	S504L	VUS		0.796	Bigger, more hydrophobic, and residue is located a highly conserved region	Cavity altered	0.417
	A: 7.96400 × 10^−6^			VUS				
				B				
rs1380306157	T: 0.999992036	Y470C	VUS	VUS	0.941	Smaller and more hydrophobic that will affect hydrogen bond formation	Cavity altered	0.879
	C: 7.96400 × 10^−6^							
rs758109437	G: 0.999952217	T430R	VUS	VUS	0.933	Bigger, charge positive, less hydrophobic and residue is located near a conserved region	Buried/exposed switch	0.637
	A: 3.98190 × 10^−5^			B				
	C: 7.96400 × 10^−6^							
rs199633613	C: 0.99993629	G393A	VUS	VUS	0.548	Bigger, more hydrophobic and mutant residue reduced flexibility	Buried/exposed switch	0.358
	G: 6.37100 × 10^−5^						Buried Glycine replaced	
rs746642521	T: 1.000	T341S	VUS	VUS	0.917	Smaller and residue is located near a conserved region	No structural damage detected	−0.598
	A: 8.894 × 10^−6^							
	C: 1.368 × 10^−6^							
rs764990697	G: 0.999	R241Q	VUS	B	0.889	Smaller, uncharged residue; potential disruption of a salt bridge	Buried charge replaced	−0.301
	T: 0.001						Buried salt bridge breakage	
rs758217844	C: 1.000	E234K	VUS	VUS	0.812	Bigger, positively charged; potential disruption of a salt bridge	Cavity altered	−0.563
	T: 4.723 × 10^−5^						Buried/exposed switch	
rs866566754	A: 1.000	V143G	VUS	VUS	0.615	Smaller, less hydrophobic and the mutation will cause loss of hydrophobic interactions in the core of the protein	Cavity altered	−2.698
	C: 4.105 × 10^−6^						Buried/exposed switch	
rs140675301	T: 0.999	E128V	B	VUS	0.031	Smaller, more hydrophobic, charge is neutral can cause loss of interactions with other molecules	No structural damage detected	−0.274
	A: 0.001							
rs2125268711	Not reported	A117V	VUS	VUS	0.453	Bigger and mutant residue located at the protein surface can disturb interactions with other molecules	No structural damage detected	0.142
				B				
rs1697316431	C: 1.000	C108S	VUS		0.887	More hydrophobic and residue is located near a highly conserved region	No structural damage detected	−0.676
	G: 2.053 × 10^−6^			VUS				
	

ACMG (American College of Medical Genetics); P = Pathogenic; VUS = Variants of Uncertain Significance; B = Benign Abbreviations; ΔΔG = Gibbs free energy change; negative values indicate reduced structural stability. MetaRNN score (0–1), reflecting pathogenicity probability. Complementary to HOPE structural analysis.

## Data Availability

The original contributions presented in this study are included in the article and Supplementary Materials. Further inquiries can be directed towards the corresponding authors.

## Supplementary Materials

The following supporting information can be downloaded at: https://www.mdpi.com/article/10.3390/genes17010072/s1, Figure S1: Comparison of ionic interactions between wild-type and mutant residues for the 13 analyzed variants. Figure S2: Comparison of ionic interactions between wild-type and mutant residues for the 7 analyzed variants of validation. Table S1: Comparative analysis of clinical and functional prediction tools for the seven validated STAT4 variants. Table S2: Comparative structural and stability assessment of the seven STAT4 variants used for validation.
