# Supplementary material for: In Silico Functional and Structural Analysis of STAT4 Variants of Uncertain Significance
Source: Genes (Basel). 2026 Jan 7;17(1):72. doi: 10.3390/genes17010072 (PMC12841567; doi:10.3390/genes17010072)
Supplement: Supplementary file 1 [file genes-17-00072-s001.zip › genes-4075213-supplementary.pdf]

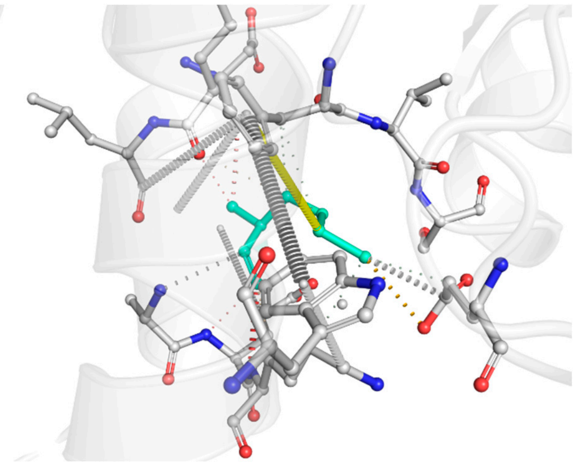

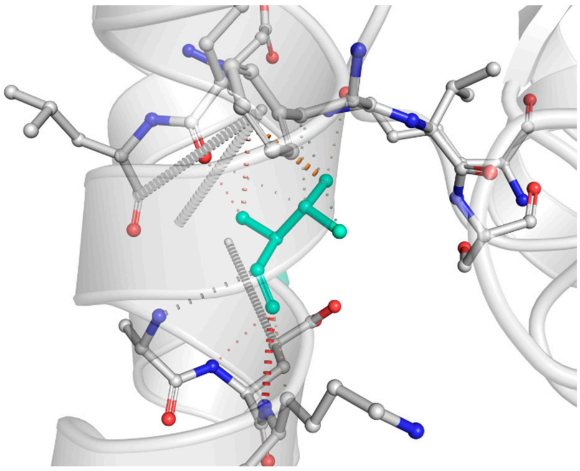

A) M517V Wild Type

B) M517V Mutant Type

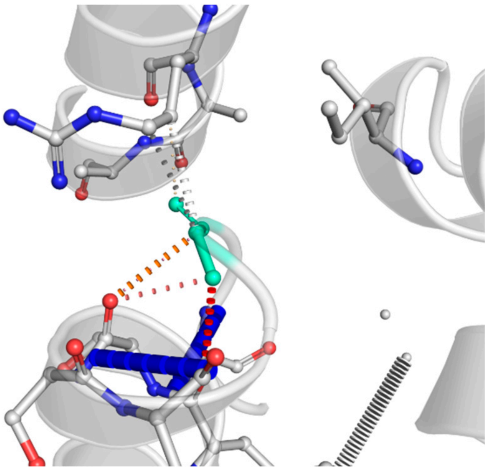

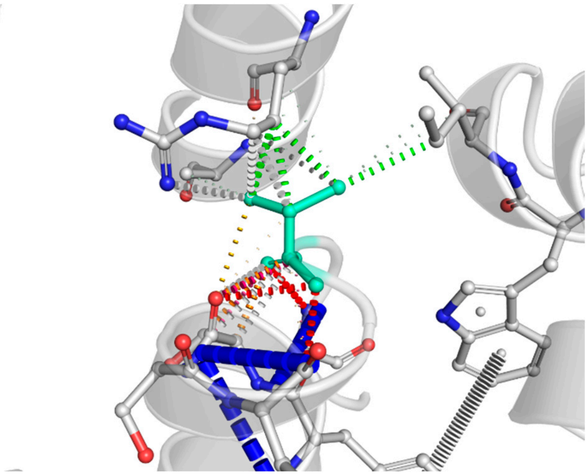

C) G507V Wild Type

D) G507V Mutant Type

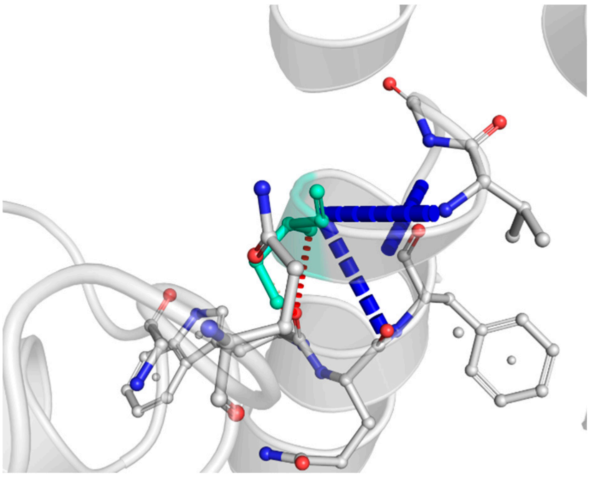

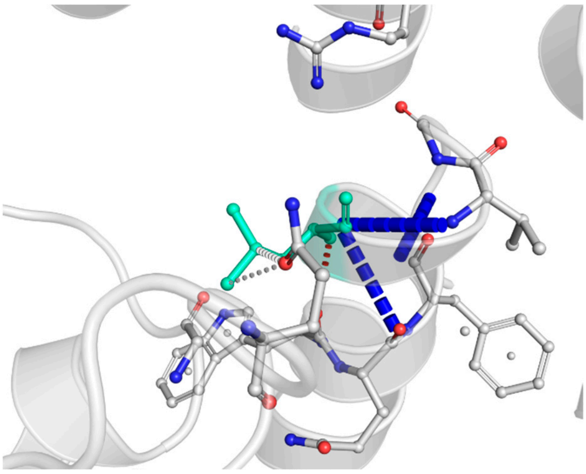

E) S504L Wild Type

F) S504L Mutant Type

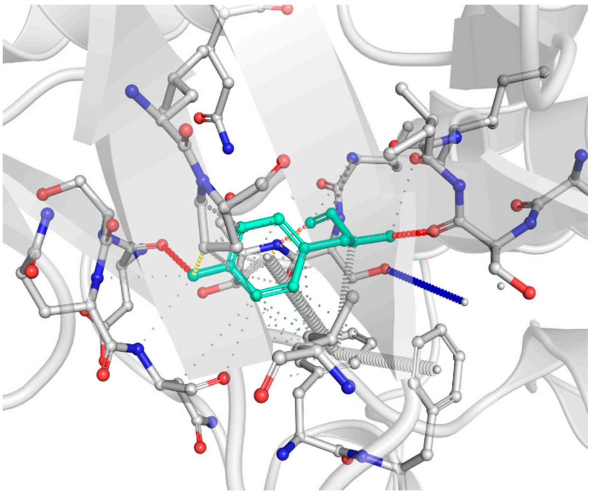

G) Y470C Wild Type

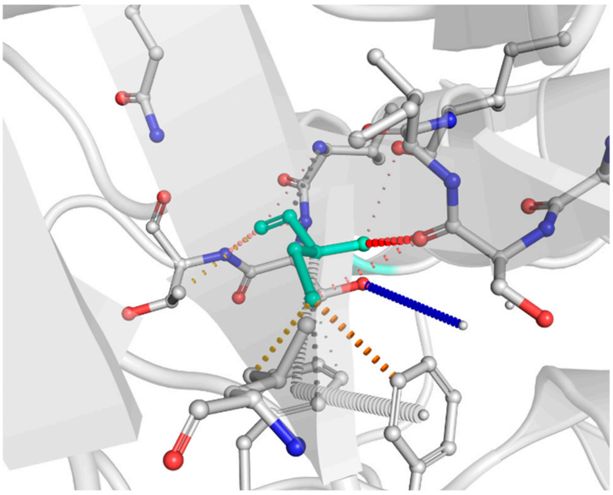

H) Y470C Mutant Type

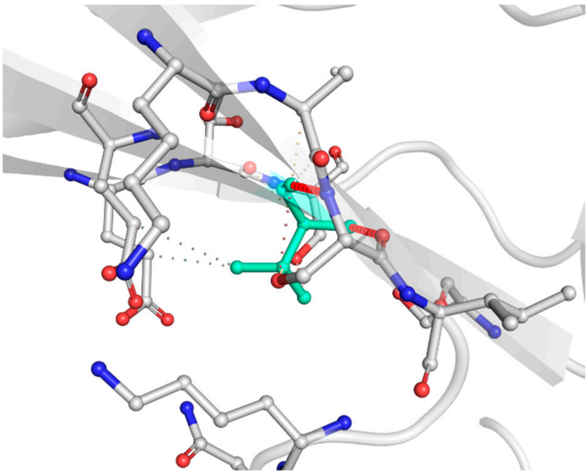

I) T430R Wild Type

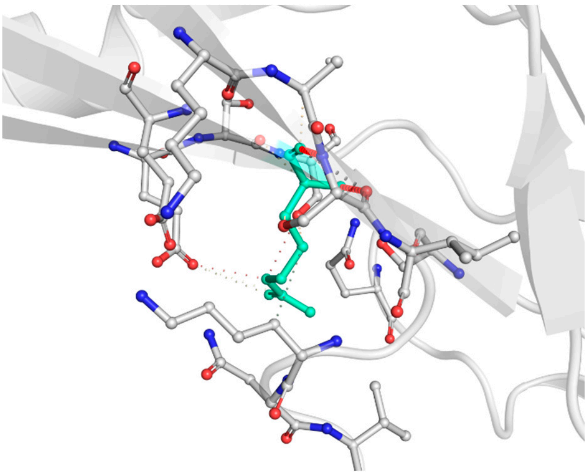

J) T430R Mutant Type

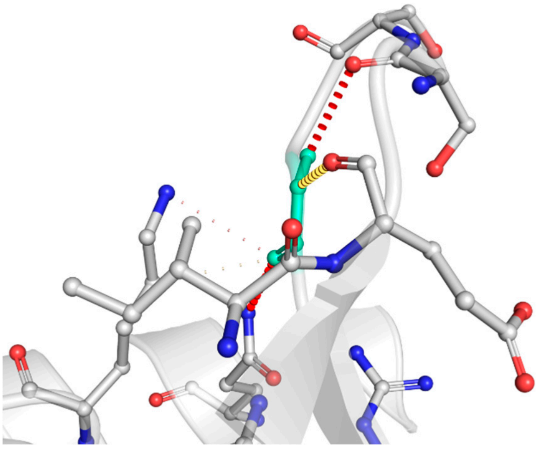

K) G393A Wild Type

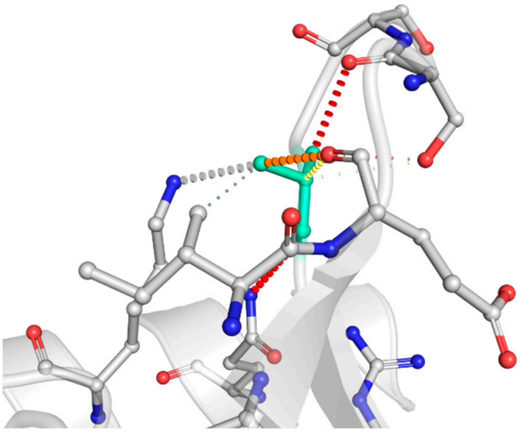

L) G393A Mutant Type

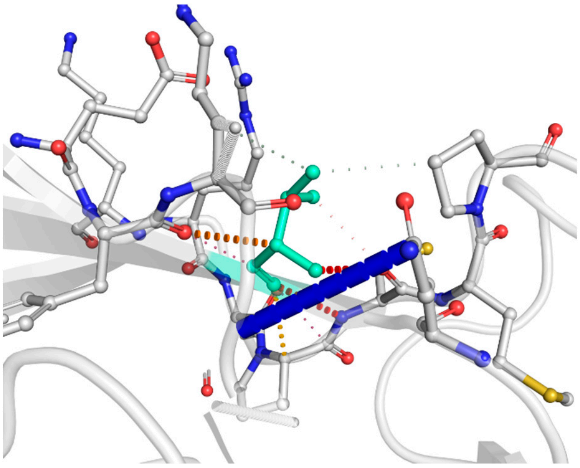

M) T341S Wild Type

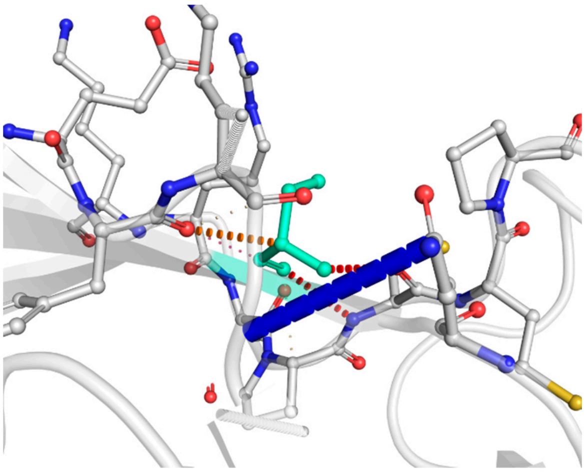

N) T341S Mutant Type

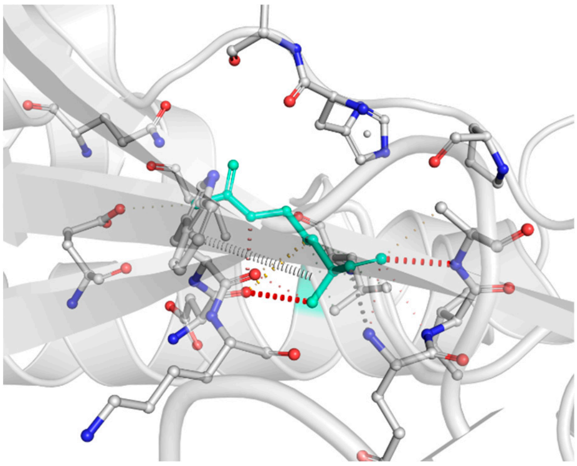

O) R241Q Wild Type

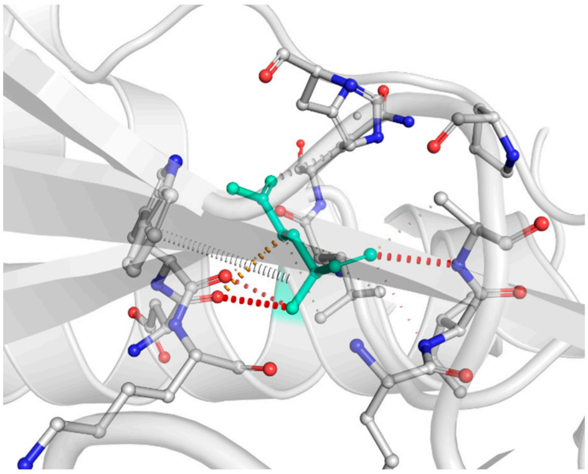

P) R241Q Mutant Type

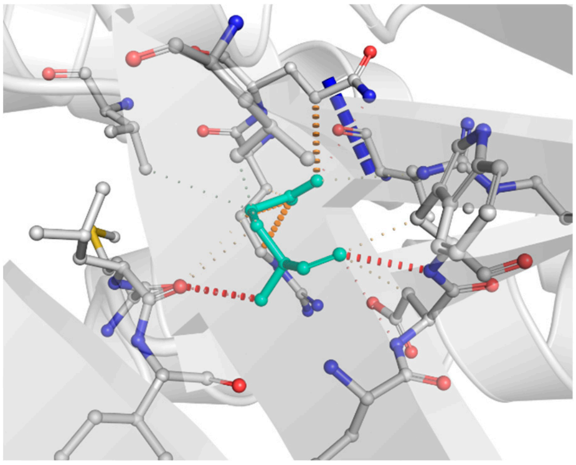

Q) E234K Wild Type

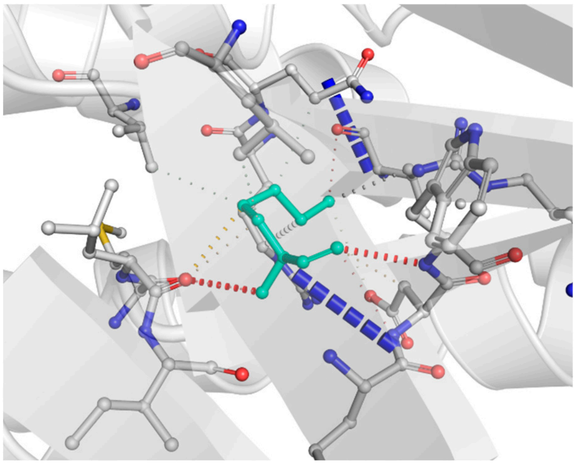

R) E234K Mutant Type

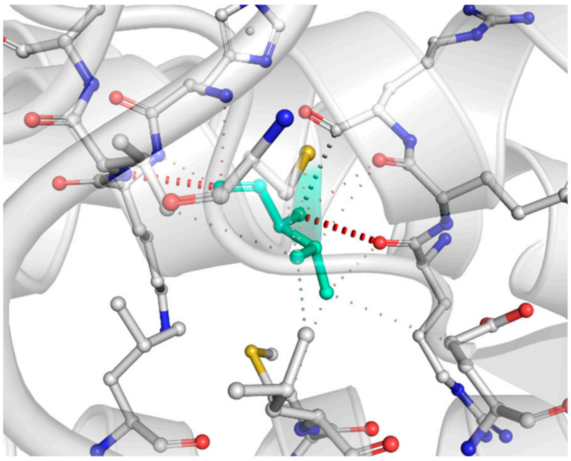

S) V143G Wild Type

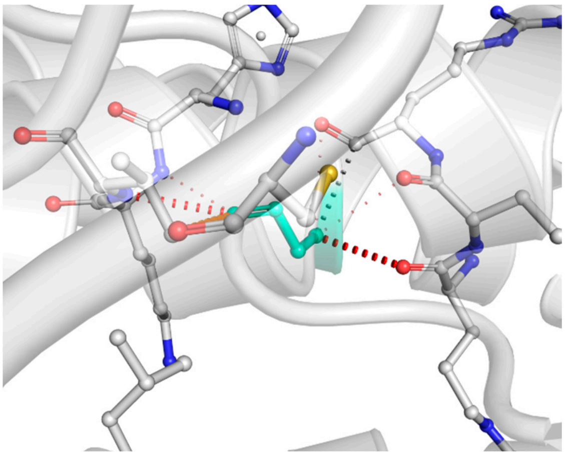

T) V143G Mutant Type

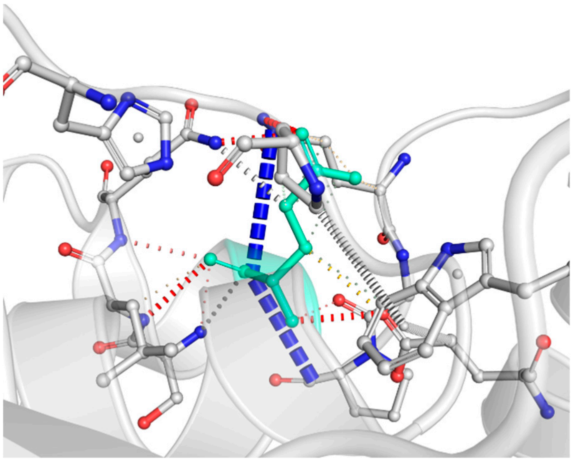

U) E128V Wild Type

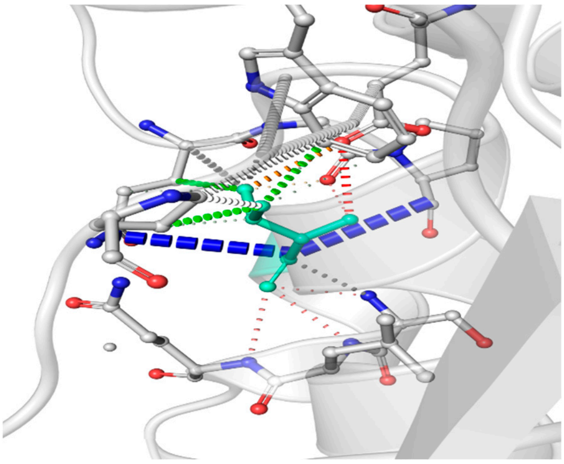

V) E128V Mutant Type

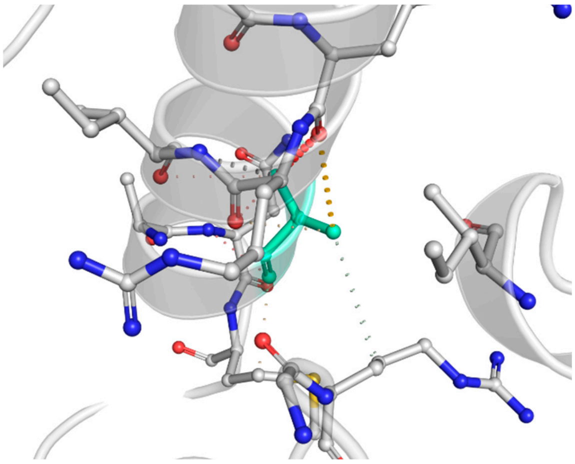

W) A117V Wild Type

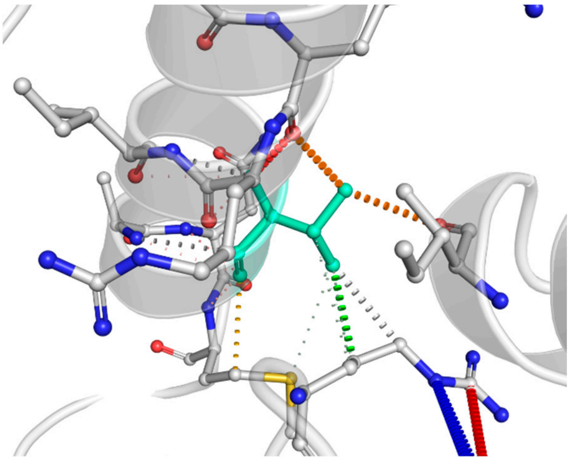

X) A117V Mutant Type

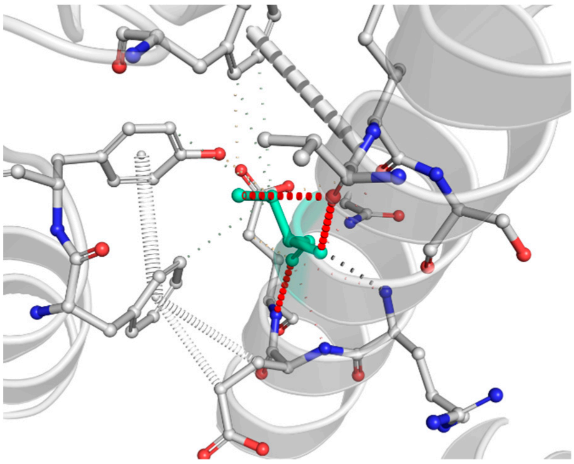

Y) C108S Wild Type

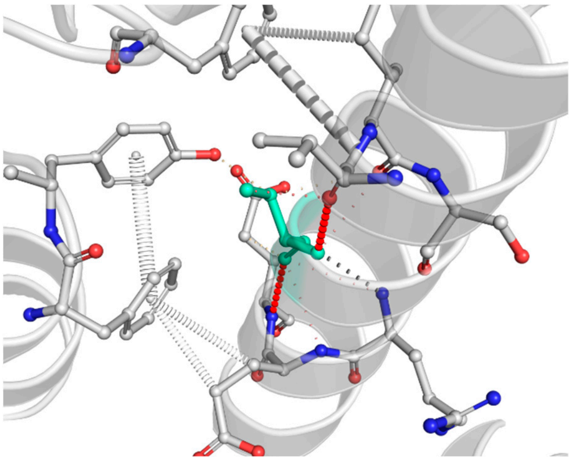

Z) C108S Mutant Type

Figure S1. Comparison of ionic interactions between wild-type and mutant residues for the 13 analyzed variants. The left panel depicts the wild-type residues, while the right panel shows the corresponding mutant residues. Each variant is labelled according to its position in the protein sequence. The letter preceding the position number indicates the wild-type amino acid, and the letter following it represents the substituted amino acid resulting from the missense mutation.

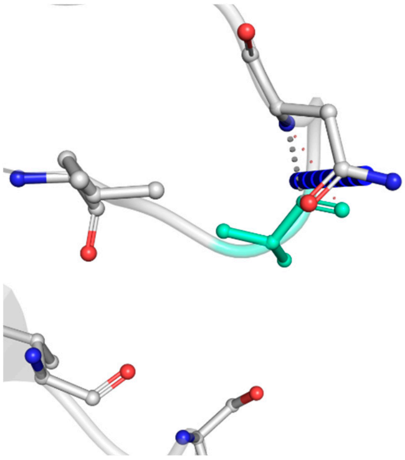

A) A650D Wild Type

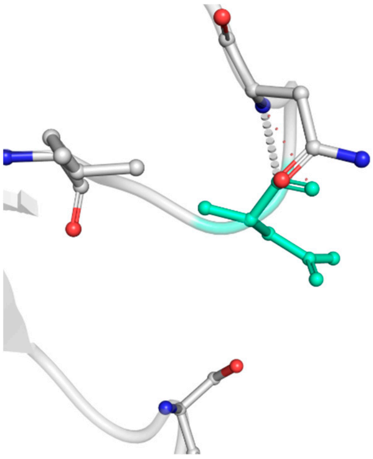

B) A650D Mutant Type

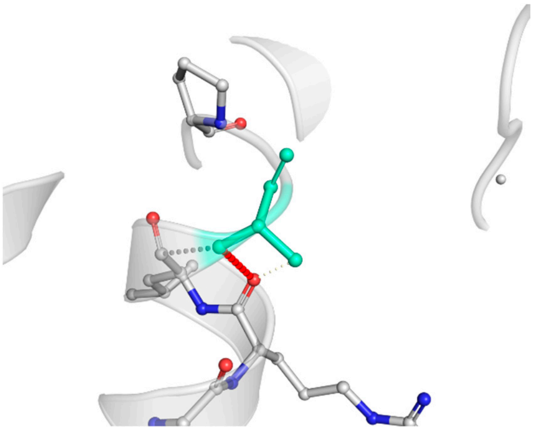

C) A635V Wild Type

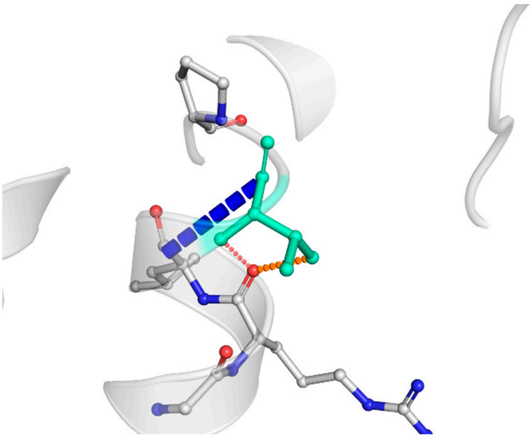

D) A635V Mutant Type

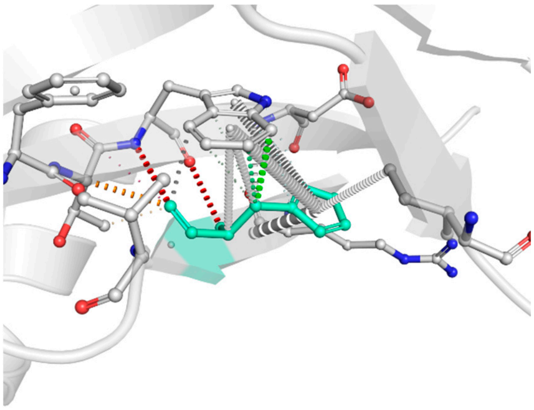

E) H623Y Wild Type

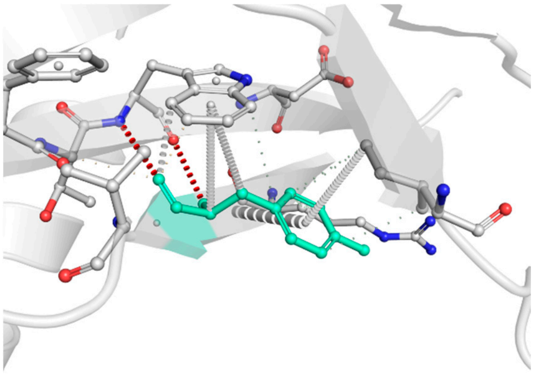

F) H623Y Mutant Type

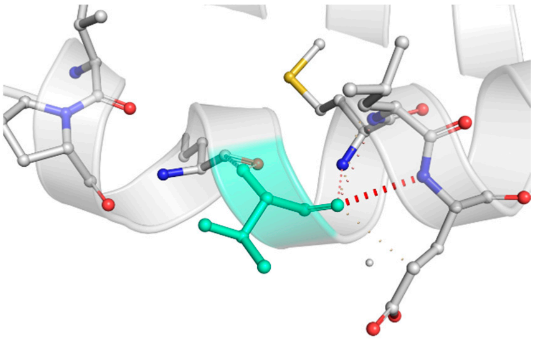

G) T298I Wild Type

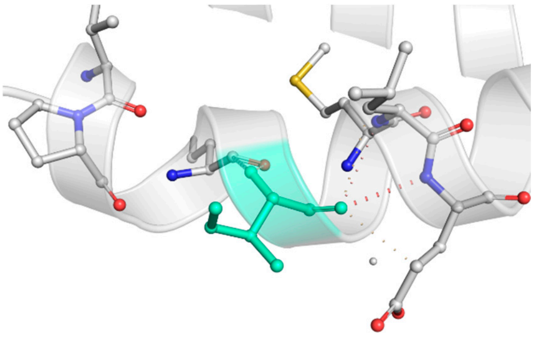

H) T298I Mutant Type

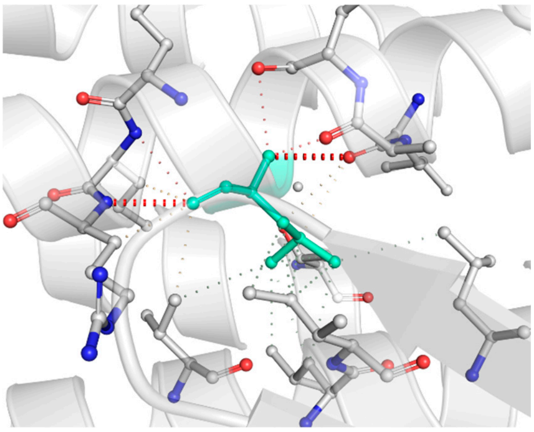

I) L269I Wild Type

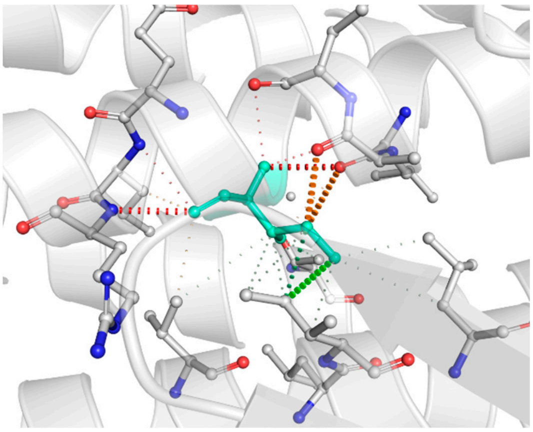

J) L269I Mutant Type

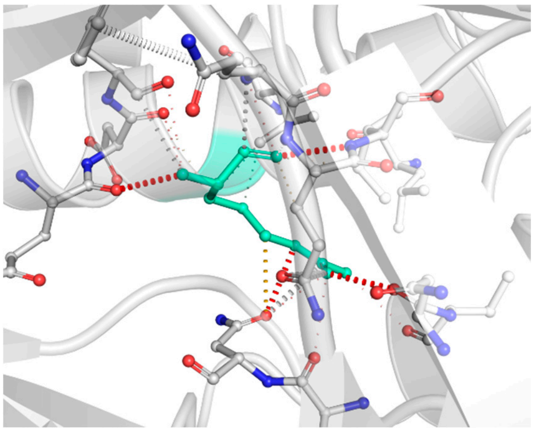

K) R240Q Wild Type

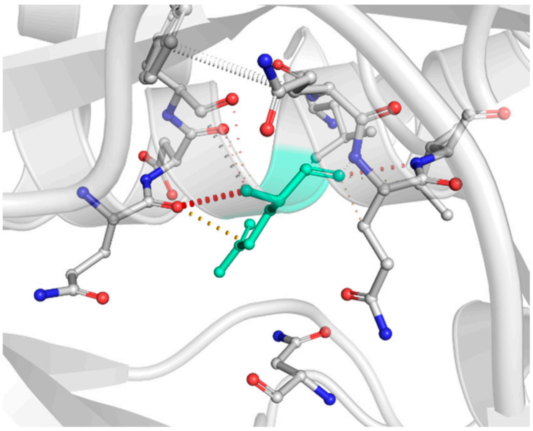

L) R240Q Mutant Type

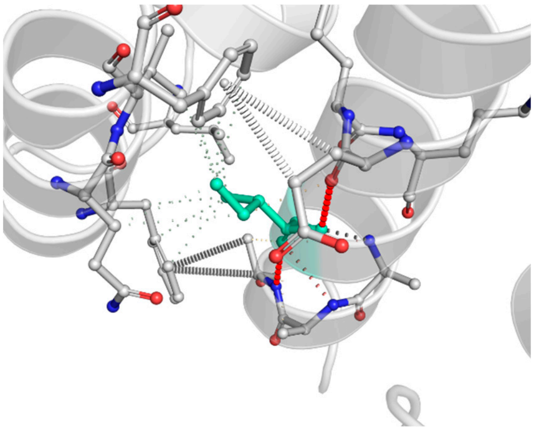

M) I115V Wild Type

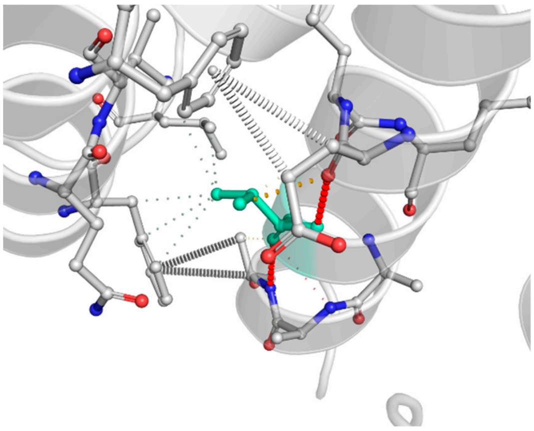

N) I115V Mutant Type

Figure S2. Comparison of ionic interactions between wild-type and mutant residues for the seven analyzed variants of validation. The left panel depicts the wild-type residues, while the right panel shows the corresponding mutant residues. Each variant is labelled according to its position in the protein sequence. The letter preceding the position number indicates the wild-type amino acid, and the letter following it represents the substituted amino acid resulting from the missense mutation.

Table S1. Comparative analysis of clinical and functional prediction tools for the seven validated *STAT4* variants

Functional and Structural Predictions of Variants

Variant	Amino Acid change	ClinVar	ACMG	VarSome	SIFT prediction	SIFT score	PolyPhen prediction	PolyPhen score
rs2470644108	A650D	P	VUS	B	deleterious	0.03	benign	0.04
rs2470644258	A635V	LP	LP	VUS	deleterious	0	possibly damaging	0.932
rs2470644386	H623Y	P	VUS	B	deleterious	0.01	possibly damaging	0.798
rs200982266	T298I	LB	LB	B	deleterious	0	benign	0.013
rs35279173	L269I	B	VUS	VUS<br>B	deleterious	0	possibly damaging	0.759
rs61756200	R240Q	LB	B	VUS<br>B	tolerated	0.1	possibly damaging	0.808
rs3024839	I115V	LB	VUS	VUS	deleterious	0.04	possibly damaging	0.807

Abbreviations: ACMG = American College of Medical Genetics and Genomics; P = Pathogenic; LP = likely pathogenic; LB = likely benign; VUS = variant of uncertain significance; B = benign.

Table S2. Comparative structural and stability assessment of the seven *STAT4* variants used for validation

Evaluation of Stability, Structural Modeling and Structural Impact of Variants in Proteins

Variant	Amino Acid change	Align GVGD	MutPred2		ERRAT	Missense 3D	HOPE		Dynamut
rs2470644108	A650D	C65	0.456	-	97.5073	-	Larger residue located within the SH2 domain; associated with altered transmembrane protein properties	0.15	0.536
rs2470644258	A635V	C55	0.571	Altered Transmembrane protein	97.5073	No structural damage detecteyd	Larger residue located within the SH2 domain; associated with altered transmembrane protein properties	0.583	-0.102
rs2470644386	H623Y	C65	0.607	Loss of Intrinsic disorder<br>Altered Ordered interface<br>Altered Transmembrane protein<br>Gain of Sulfation at H623	97.3568	No structural damage detected	Larger, more hydrophobic residue located within the SH2 domain; predicted loss of intrinsic disorder, altered ordered interface, altered transmembrane region, and gain of sulfation at H623	0.483	0.557
rs200982266	T298I	C65	0.092	-	97.5073	No structural	Bigger and more hydrophobic residue	0.014	-0.38

						damage detected	located in a domain important for protein activity and in contact with another domain		
rs35279173	L269I	C0	0.318	-	97.5073	No structural damage detected	Bigger and will not fit in the core of the protein	0.019	0.417
rs61756200	R240Q	C35	0.533	Altered Coiled coil Altered Transmembrane protein	97.5073	No structural damage detected	Smaller, neutral residue possibly causing loss of external interactions; altered coiled-coil and transmembrane regions	0.055	-1.741
rs3024839	I115V	C25	0.393	-	97.5073	No structural damage detected	Smaller residue located within a conserved region	0.011	-1.009

Abbreviations: ΔΔG = Gibbs free energy change (negative values indicate reduced structural stability);. MetaRNN score (0–1) reflects pathogenicity probability; results are complementary to HOPE structural analysis.
